# Raman-assisted broadband mode-locked laser

**DOI:** 10.1038/s41598-019-40313-2

**Published:** 2019-03-06

**Authors:** Shota Kimura, Shuntaro Tani, Yohei Kobayashi

**Affiliations:** The Institute for Solid State Physics, The University of Tokyo, 5-1-5 Kashiwanoha, Kashiwa, Chiba 277-8581 Japan

## Abstract

The pulse duration that is available from femtosecond mode-locked lasers is limited by the emission bandwidth of the laser crystals used. Considerable efforts have been made to broaden the emission gain bandwidth in these lasers over the past five decades. To break through this limitation, intracavity spectral broadening is required. Here, we propose a new spectral broadening method inside the mode-locked cavity based on use of stimulated Raman scattering and demonstrate significant pulse shortening using this method. We configured Kerr-lens mode-locked lasers based on Yb:CaGdAlO_4_, Yb:KY(WO_4_)_2_ and Yb:Y_2_O_3_ materials and achieved significant spectral broadening that exceeds the emission bandwidth. The spectral broadening in the Yb:CaGdAlO_4_ oscillator shortens the pulse duration to 22 fs, which is a one-third of the duration of our unbroadened mode-locked pulse. The results presented here indicate that Raman-assisted spectral broadening can break the limitations of the emission gain bandwidth and shorten the duration of pulses from femtosecond mode-locked lasers.

## Introduction

Ultra-short pulse laser oscillators have been studied for the last half-century. To date, passive mode locking methods such as Kerr-lens mode locking have been used to generate sub-5-fs pulses^1–4^. However, progress in shortening of the pulse duration in a laser oscillator has remained stagnant over the last two decades because almost the full bandwidths of the available laser material gains had already been used. Therefore, to realize shorter pulse durations beyond the spectral width of the stimulated emission gain, other gain schemes must be explored inside the laser oscillator itself.

**Figure 1:**
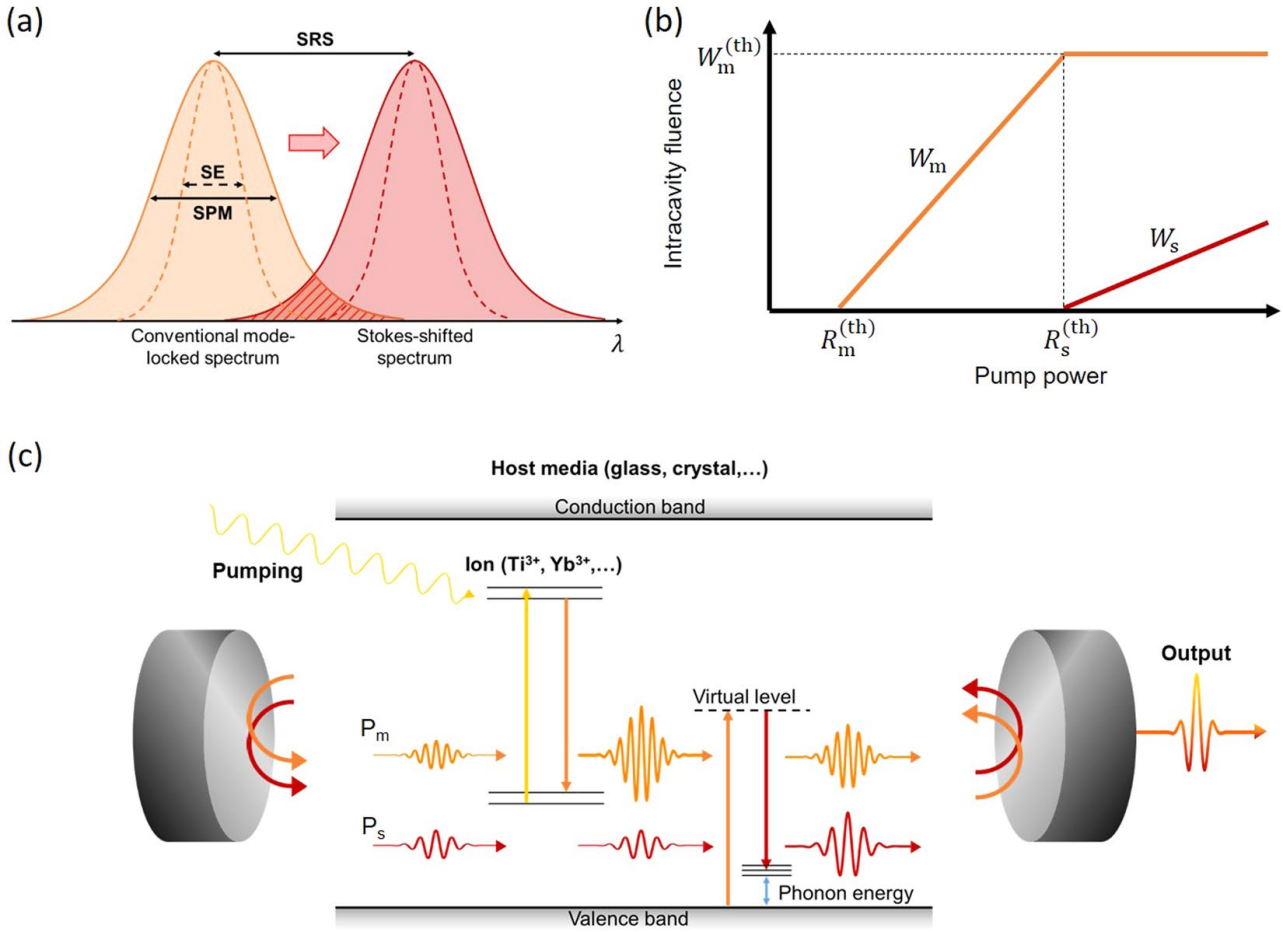
Concept of Raman-assisted broadband mode-locked laser. (**a**) Characteristics of Raman spectrum induced by a mode-locked laser pulse. The two spectra have a spectral overlap because of the broad spectrum of the fundamental mode-locked pulse. SE: stimulated emission; SPM: self-phase modulation; SRS: stimulated Raman scattering. (**b**) Analytical solutions to the master equations depicted schematically. W_m_ and W_s_ represent the intracavity fluences of P_m_ and P_s_, respectively. There are two pump power thresholds: R_m_^(th)^ and R_s_^(th)^. A Stokes-shifted pulse appears when the intracavity fluence of W_m_ exceeds the threshold fluence W_m_^(th)^. W_s_ is proportional to the pump power close to the threshold. (**c**) Conceptual illustration of the Raman-assisted broadband mode-locked laser. Orange arrows represent the fundamental mode-locked pulse (P_m_) and red arrows represent the Stokes-shifted pulse (P_s_) induced by SRS. In the laser crystals, P_m_ receives gain from an inverted population of doped ions that are excited by higher energy photons (yellow arrow), while P_s_ does not receive any gain or loss from doped ions because of the low photon energy. In contrast, P_s_ receives Raman gain from P_m_ throughout the host medium. The wavelength of P_s_ is determined by the phonon energy of the host medium. Output pulses are given by the superposition of P_m_ and P_s_.

One candidate method for such an intracavity gain scheme involves use of third-order nonlinear optical processes, which have been used to convert laser spectra in cavities for five decades^5–12^. Specifically, both self-phase modulation (SPM) and stimulated Raman scattering (SRS) can be used to broaden and shift laser spectra by several tens of terahertz^13^. SPM has played an important role in ultrashort pulse generation and the associated spectral broadening^2,8,14,15^. SRS has also been used to extend the spectral coverage of laser sources in the cavity^16^. Synchronously pumped Raman lasers have been realized to generate femtosecond laser pulses^17–19^. However, SRS has not been regarded as a viable gain scheme for ultrashort pulse generation in mode-locked oscillators because of several associated difficulties, including the coexistence of both a laser pulse and a Stokes-shifted pulse, and the required adjustment of the phase relationship between the laser pulse and the Stokes-shifted pulse. Recently, Babin *et al*. demonstrated a mode-locked fiber oscillator, in which a separate Raman gain coexists inside the cavity^20^. The optical phase locking between the laser pulse and the Stokes-shifted pulses has been remaining issue to achieve a shorter pulse generation supported by SRS. If SRS can be used in cooperation with SPM in a cavity, game-changing spectral broadening may then be possible because the magnitude of the Stokes shift becomes comparable to the spectral width of the Kerr-lens mode-locked pulses, meaning that the spectral width can then be multiplied, as shown in Fig. 1(a).

Here, we propose a scheme for spectrum multiplication in this manner that uses SRS with Kerr-lens mode locking in an oscillator to generate ultrashort pulses. In our scheme, a pulse that is generated via Kerr-lens mode locking, which we refer to as the main pulse in this paper, induces SRS to generate a Stokes-shifted pulse. The oscillator is designed to induce Kerr-lens mode-locking so that the laser oscillation becomes stable when the intracavity pulse duration becomes shorter, resulting in phase locking over the entire spectrum. The magnitude of the Stokes shift is selected to match the spectral width of the main pulse to cause the two spectra to overlap. The overlapped spectrum induces a frequency pulling phenomenon between the main pulse and the Stokes-shifted pulse, which then realizes phase locking over the entire spectrum. This phenomenon causes pulse shortening in the time domain.

## Results

### Criterion of Raman-assisted spectrum multiplication

One key issue for realization of this scheme is that the Raman gain is rather weak when compared with the stimulated emission gain^13^. For the purposes of spectrum multiplication, the output spectra of the two pulses from the oscillator should be comparable. We therefore derived the criterion for spectral multiplication using the energy master equation for a mode-locked laser (see Methods):

1 $${W}_{{\rm{m}}}^{({\rm{th}})}=\frac{8}{\pi }\frac{{\tau }_{{\rm{ave}}}}{{g}_{{\rm{R}}}d}{l}_{{\rm{s}}},$$

where ${W}_{{\rm{m}}}^{({\rm{th}})}$ is the threshold fluence of the main pulse that originated from the stimulated emission gain, *l*_s_ is the cavity loss of the Stokes-shifted pulse, *g*_R_ is the Raman gain coefficient, *d* is the interaction length of the Raman medium and ${\tau }_{{\rm{ave}}}$ is the average pulse duration of both the main pulse and the Stokes-shifted pulse. Figure 1(b) shows the calculated intracavity fluences of both the main pulse ${W}_{{\rm{m}}}$ and the Stokes-shifted pulse ${W}_{{\rm{s}}}$ as functions of the pump power. When the intracavity fluence of the main pulse ${W}_{{\rm{m}}}$ satisfies the condition that ${W}_{{\rm{m}}}\,\geqq \,{W}_{{\rm{m}}}^{({\rm{th}})}$ with increasing pump power, the Raman gain then overcomes the cavity loss and generates the Stokes-shifted pulse. During this process, extra pump power is efficiently converted into the Stokes-shifted pulse, thus ensuring that ${W}_{{\rm{m}}}$ remains at ${W}_{{\rm{m}}}^{({\rm{th}})}$. While this extra pump power can only enhance the main pulse, the resulting enhanced pulse transfers energy to the Stokes-shifted pulse until the Raman gain and the cavity loss equilibrate, as shown in Fig. 1(c).

From Eq. (1), we found that the lower loss and the shorter pulse duration were both crucial to reduction of the required pulse fluence ${W}_{{\rm{m}}}^{({\rm{th}})}$, which meant that a high-Q cavity was desirable for activation of the proposed spectrum multiplication mechanism. The threshold intracavity fluence can be estimated to be ${W}_{{\rm{m}}}^{({\rm{th}})} \sim 10\,{\rm{mJ}}/{{\rm{cm}}}^{2}$ in a high-Q cavity (${l}_{s}=1 \%$) that has a small Raman gain coefficient $({g}_{R}=0.01\,{\rm{cm}}/{\rm{GW}}$), uses a 1-mm-thick crystal and has ${\tau }_{{\rm{ave}}}=100\,{\rm{fs}}$.

### Demonstration of spectrum multiplication and ultrashort pulse generation

**Figure 2:**
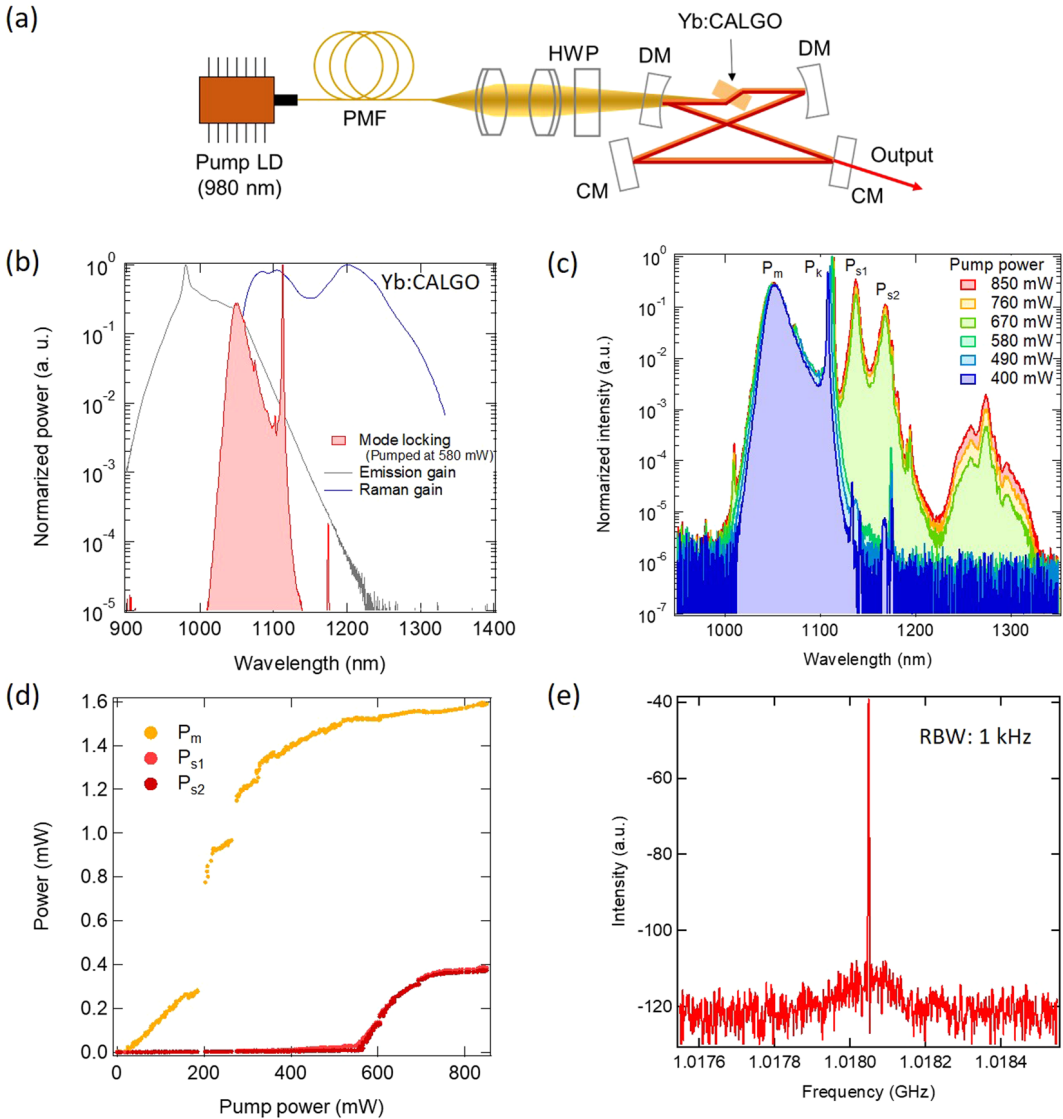
Spectral properties of the Yb:CALGO Kerr-lens mode-locked laser. (**a**) Experimental setup. PMF: polarisation-maintaining fibre; HWP: half-wave plate; DM: dichroic mirror; CM: chirp-compensating mirror. (**b**) Optical spectrum of mode-locked pulses (red) pumped at 580 mW shown with the emission gain spectrum (gray) and the Raman gain spectrum (blue), which was calculated as the convolution of the Raman spectrum and the mode-locked spectrum. (**c**) Optical spectra at various pump powers. P_m_, P_k_, P_s1_ and P_s2_ denote the peaks occurring at 1048 nm, 1112 nm, 1137 nm and 1167 nm, respectively. (**d**) Pump power dependences of output power-integrated and calibrated optical spectra. P_m_ (yellow), P_s1_ (red) and P_s2_ (dark red) are plotted. (**e**) RF spectrum at the pump power of 850 mW shown in (**c**) RBW: resolution bandwidth.

To demonstrate both spectrum multiplication and ultrashort pulse generation with SRS, we configured a high-Q Kerr-lens mode-locked laser using a Yb:CaGdAlO_4_ (CALGO) crystal, as shown in Fig. 2(a). We selected the combination of Yb and CALGO for the following reason: while we can choose a wide variety of host media for use in Yb-doped materials, the Yb:CALGO crystal can generate a 32 fs pulse because of the broad emission gain, which makes it suitable for spectral broadening applications^21^. In addition, the tetragonal structure of the CALGO crystal provides a broad Raman gain because of its complexity. Figure 2(b) shows the mode-locked spectrum obtained at a pump power of 580 mW with normalized emission and Raman gain spectra. The Raman gain spectrum was calculated as the convolution of the Raman spectrum and a mode-locked spectrum (see Supplementary Information). The transform-limited pulsed duration of this mode-locked spectrum was 67 fs at the 580 mW pump power. The narrower side peak was determined to be a Kelly sideband^22^ (see Supplementary Information).

Figure 2(c) illustrates the pump-power dependence of the optical spectrum. The main mode locking peak (P_m_) showed gradual spectral broadening with increasing pump power. When the pump power was increased to 850 mW, sudden spectral broadening was observed from 1100 nm to 1200 nm. The spectral bandwidth at the 850 mW pump power level corresponded to a pulse duration of 19 fs at the Fourier transform limit. Two prominent peaks were found at wavelengths of 1137 nm (P_s1_) and 1167 nm (P_s2_). The output power at each peak is shown as a function of the pump power in Fig. 2(d). P_s1_ and P_s2_ showed threshold-like behaviour at the threshold pump power of approximately 600 mW. The powers of both P_s1_ and P_s2_ showed linear increases near this threshold. This behaviour agrees well with our theoretical analysis when SRS is included (Fig. 1b). The wavelengths of these two peaks were largely determined by the cavity dispersion (See Supplementary Information). Figure 2(e) shows the radio-frequency (RF) spectrum measured using an InGaAs photodetector at the pump power of 850 mW shown in Fig. 2(c). A clear and narrow peak was observed at a frequency of 1.0180 GHz without any sidebands. This result means that both the main pulse and the Stokes-shifted pulse are well mode-locked at the same repetition rate.

**Figure 3:**
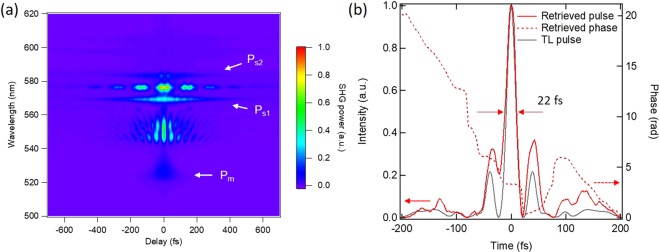
FROG trace of Yb:CALGO Kerr-lens mode-locked laser. (**a**) A raw SHG-FROG trace at the pump power of 850 mW shown in Fig. 2(c). (**b**) Temporal profile identified using a FROG measurement. The gray line shows the transform limited pulse. The FWHM duration of the transform limited pulse is 19 fs. The red line indicates a retrieved temporal profile. The FWHM duration is 22 fs. The red dash line shows a retrieved phase.

Because of the Kerr-lens mode locking mechanism used in the cavity, the entire spectrum, combining the main peak (P_m_) and the Raman peaks (P_s1_, P_s2_), should generate the ultrashort pulses. We performed a second-harmonic generation frequency-resolved optical gating (SHG-FROG) measurements to evaluate the temporal profile of the pulse after compensating for the chirp of the output pulse using a pair of SF6 prisms. The raw SHG-FROG trace is shown in Fig. 3(a). The SHG signal and sum frequency generation (SFG) caused by the main peak (P_m_) and the Raman peaks (P_s1_, P_s2_) were clearly observed. However, the SHG signal that originated from the Kelly sideband (P_k_) was not detected because of its long pulse duration (>0.9 ps). Figure 3(b) shows a retrieved temporal profile. The pulse has temporal sidebands that originate from a beat between the main peak (P_m_) and the Stokes-shifted peaks (P_s1_, P_s2_), as shown in Fig. 3(a). The full width at half maximum (FWHM) duration of the pulse was 22 fs, which is close to the Fourier transform limit. This result verifies mode locking of the entire spectrum, including both the main peak (P_m_) and the Raman peaks (P_s1_, P_s2_).

### Universality of spectrum multiplication

**Figure 4:**
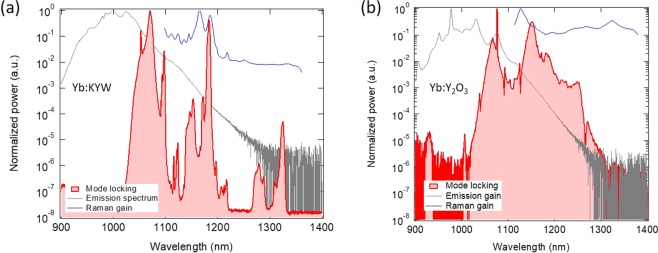
Universality of spectral broadening. (**a**) Optical spectrum of Yb:KYW mode-locked laser (red) with emission gain spectrum (gray) and Raman gain spectrum (blue). (**b**) Optical spectrum of Yb:Y_2_O_3_ ceramic mode-locked laser (red) with emission gain spectrum (gray) and Raman gain spectrum (blue).

To verify the universality of the proposed mechanism, we also constructed other high-Q oscillators using two other host media: a Yb:KY(WO_4_)_2_ (KYW) crystal and a Yb:Y_2_O_3_ ceramic. Figure 4 shows the output spectra of these mode-locked lasers under sufficiently strong pumping to satisfy the condition that ${W}_{{\rm{m}}}\,\geqq \,{W}_{{\rm{m}}}^{({\rm{th}})}$. In both the Yb:KYW and Yb:Y_2_O_3_ lasers, we observed the typical mode-locked spectrum at a wavelength of approximately 1070 nm and the same spectral broadening from 1100 nm to 1200 nm with significant peaks. These results for the different host media with the high-Q cavity demonstrated the universality of the SRS spectral broadening concept for use in mode-locked oscillators.

## Discussion

The proposed spectral multiplication technique has the potential to spread the use of our method to a variety of ultrafast laser oscillators. Appropriate cavity design (including dispersion, Q-factor, and repetition rate) may trigger cascaded SRS, which would result in the spectral width being multiplied by several times. Our phase-locked spectral broadening scheme could be applied not only to oscillators but also to amplifiers, leading to higher power generation. Further pulse duration shortening could be achieved by designing appropriate Raman gain and shift properties in the laser gain medium.

In conclusion, we have demonstrated a Raman-assisted broadband mode-locked laser using a high-Q cavity design. A theoretical study was performed to derive the criterion for spectral broadening when using SRS. A Yb:CALGO mode-locked laser showed a broader output spectrum and a pulse duration of 22 fs was achieved using this laser. The universality of the spectral broadening concept was confirmed using both a Yb:KYW crystal and a Yb:Y_2_O_3_ ceramic with the high-Q cavity. To the best of our knowledge, this is the first demonstration of a Raman-assisted broadband mode-locked laser. Our analytical and experimental results open the way towards engineering of a new ultrafast mode-locked laser.

## Methods

### Master equations

The master equation for a mode-locked pulse was originally derived by Haus^23^. We added the SRS effect to this master equation and derived the following fluence rate equations for two mode-locked pulses (See Supplementary Information):

2a $${T}_{{\rm{R}}}\frac{d}{dT}{W}_{{\rm{m}}}(T)=[{g}_{{\rm{m}}}-{l}_{{\rm{m}}}-\frac{\pi }{8}\frac{{\lambda }_{{\rm{s}}}}{{\lambda }_{{\rm{m}}}}\frac{{g}_{{\rm{R}}}d}{{\tau }_{{\rm{ave}}}}\,{W}_{{\rm{s}}}(T)]{W}_{{\rm{m}}}(T),$$

2b $${T}_{{\rm{R}}}\frac{d}{dT}{W}_{{\rm{s}}}(T)=[-{l}_{{\rm{s}}}+\frac{\pi }{8}\frac{{g}_{{\rm{R}}}d}{{\tau }_{{\rm{ave}}}}\,{W}_{{\rm{m}}}(T)]{W}_{{\rm{s}}}(T).$$

where *T* is the time scale of pulse evolution during a cavity round trip, *T*_R_ is the round-trip time and ${\lambda }_{{\rm{i}}}$ denotes each wavelength (where ${\rm{i}}={\rm{m}},{\rm{s}}$). In the steady-state, the emission gain can be described simply by ${g}_{{\rm{m}}}=R/({W}_{{\rm{m}}}+1/\sigma {\tau }_{{\rm{inv}}})$ using a rate equation for population inversion, where *R* is the pumping rate, $\sigma$ is the emission cross-section and ${\tau }_{{\rm{inv}}}$ is the population inversion lifetime^13^. Substitution of *g*_m_ into Eq. (2a) allows the steady-state solution to be derived analytically, as shown in Fig. 1(b). The analytical solutions are described in the Supplementary Information.

### Lasers

Two concave mirrors (radius *r* = 15 mm) and two plane mirrors were used to configure the bow-tie ring cavity. All mirrors had high reflectivity of more than 99.7% over the range from 1020 nm to 1170 nm to increase intracavity intensity. Both plane mirrors were chirp-compensating mirrors, and one of these mirrors was used as an output coupler with transmittance of 0.02%. A 1-mm-thick, c-cut, uncoated 3 at.% Yb:CALGO crystal was placed between the concave mirrors at the Brewster angle. The pump source was a wavelength-stabilized 980 nm laser diode that was coupled using a polarisation-maintaining fibre. The output power was 3 mW with a repetition rate of 1 GHz.

The optical components of both the Yb:KYW and Yb:Y_2_O_3_ lasers are similar to those of the Yb:CALGO laser. The differences described as follows. In the setup for the first laser, a 2-mm-thick, 5 at.% doped Yb:KYW crystal was used with a 20-mm-radius concave mirror for the cavity. The output power was 20 mW at a repetition rate of 0.8 GHz. A 1-mm-thick, 3 at.% doped Yb:Y_2_O_3_ ceramics was used in the setup for the second laser. The output power was 50 mW at a repetition rate of 0.8 GHz.

## Supplementary information

Supplementary information

## References

[1] Rausch S (2008). Controlled waveforms on the single-cycle scale from a femtosecond oscillator. Opt. Express.

[2] Ell R (2001). Generation of 5-fs pulses and octave-spanning spectra directly from a Ti: sapphire laser. Opt. lett..

[3] Sutter DH (1999). Semiconductor saturable-absorber mirror–assisted Kerr-lens mode-locked Ti: sapphire laser producing pulses in the two-cycle regime. Opt. Lett..

[4] Morgner U (1999). Sub-two-cycle pulses from a Kerr-lens mode-locked Ti: sapphire laser. Opt. Lett..

[5] Mocker HW, Collins RJ (1965). Mode competition and self-locking effects in a Q-switched ruby laser. Appl. Phys. Lett..

[6] DeMaria AJ, Stetser DA, Heynau H (1966). Self mode-locking of lasers with saturable absorbers. Appl. Phys. Lett..

[7] Ippen EP, Shank CV, Dienes A (1972). Passive mode locking of the cw dye laser. Appl. Phys. Lett..

[8] Spence DE, Kean PN, Sibbett W (1991). 60-fsec pulse generation from a self-mode-locked Ti: sapphire laser. Opt. Lett..

[9] Woodbury EJ, Ng WK (1962). Ruby laser operation in near IR. Proc. IRE..

[10] Eckhardt G (1962). Stimulated Raman scattering from organic liquids. Phys. Rev. Lett..

[11] Vahala KJ (2003). Optical microcavities. Nature.

[12] Yang QF, Yi X, Yang KY, Vahala K (2017). Stokes solitons in optical microcavities. Nat. Phys..

[13] Yariv, A. Quantum electronics, 3rd ed. (John Wiley & Sons, New York, 1989).

[14] Salin F, Squier J, Piche M (1991). Mode locking of Ti: Al_2_O_3_ lasers and self-focusing: a Gaussian approximation. Opt. Lett..

[15] Duling IN (1991). Subpicosecond all-fibre erbium laser. Electron. Lett..

[16] Pask HM (2003). The design and operation of solid-state Raman lasers. Prog. Quant. Electron..

[17] Kafka JD, Baer T (1987). Fiber Raman soliton laser pumped by a Nd: YAG laser. Opt. Lett..

[18] Lin J, Spence DJ (2016). 25.5 fs dissipative soliton diamond Raman laser. Optics Lett..

[19] Churin D (2015). High-power synchronously pumped femtosecond Raman fiber laser. Optics Lett.

[20] Babin SA (2014). Multicolour nonlinearly bound chirped dissipative solitons. Nat. Commun..

[21] Sévillano P, Georges P, Druon F, Descamps D, Cormier E (2014). 32-fs Kerr-lens mode-locked Yb: CaGdAlO 4 oscillator optically pumped by a bright fiber laser. Opt. Lett..

[22] Kelly SMJ (1992). Characteristic sideband instability of periodically amplified average soliton. Electron. Lett..

[23] Haus HA (1975). Theory of mode locking with a fast saturable absorber. J. Appl. Phys..

