# Supplementary material for: Raman-assisted broadband mode-locked laser
Source: Sci Rep. 2019 Mar 6;9:3738. doi: 10.1038/s41598-019-40313-2 (PMC6403213; doi:10.1038/s41598-019-40313-2)
Supplement: Supplementary file 1 — Supplementary information [file 41598_2019_40313_MOESM1_ESM.docx]

**Supplementary Information**

**Raman-assisted broadband mode-locked laser**

Shota Kimura, Shuntaro Tani and Yohei Kobayashi^*^

*The Institute for Solid State Physics, The University of Tokyo, 5-1-5 Kashiwanoha, Kashiwa, Chiba 277-8581, Japan*

*e-mail: yohei@issp.u-tokyo.ac.jp

**S1. Master equations**

The master equation for a mode-locked pulse was originally derived by Haus [23]. We treat the two master equations required for the main pulse (P_m_) and the Stokes-shifted pulse (P_s_), which both include the SRS effect, as follows:

$$T_{R}\frac{\partial}{\partial T}a_{m}\left( T, t \right)=\left[ \hat{F}\left( a_{m} \right)-j\frac{\lambda_{s}}{\lambda_{m}}\delta_{x}\left| a_{s}\left( T,t \right) \right|^{2}-\frac{\lambda_{s}}{\lambda_{m}}\delta_{R}\left| a_{s}\left( T,t \right) \right|^{2} \right]a_{m}\left( T, t \right), (S1a)$$

$$T_{R}\frac{\partial}{\partial T}a_{s}\left( T, t \right)=\left[ \hat{F}\left( a_{s} \right)-j\delta_{x}\left| a_{m}\left( T,t \right) \right|^{2}+\delta_{R}\left| a_{m}\left( T,t \right) \right|^{2} \right]a_{s}\left( T, t \right). (S1b)$$

where $T_{R}$ is the round-trip time, $a_{i} (i=m,s)$ is the normalized electric field amplitude of each pulse, $\lambda_{i}$ is the wavelength of each pulse, and $\delta_{x}$ and $\delta_{R}$ are the cross-phase modulation (XPM) and the Raman coefficient, respectively. Here, we consider two time scales: $T$ is the pulse evolution time scale during a single cavity round trip, and $t$ is the time scale for evolution of the pulse envelope function. The first term on the right-hand side ($\hat{F}$) represents conventional mode-locking. The operator $\hat{F}$ is defined in ref [S1], and consists of the emission gain ($g_{i}$), the loss ($l_{i}$), the dispersion, the self-amplitude modulation (SAM) and the self-phase modulation (SPM). Here, the emission gain and loss are both assumed to be frequency-independent, and the emission gain of the Stokes-shifted pulse is set to zero. Based on these assumptions, $\hat{F}$ can then be written as

$$\hat{F}\left( a_{m} \right)=\frac{1}{2}\left( g_{m}-l_{m} \right)+jD_{m}\frac{\partial^{2}}{\partial t^{2}}-j\delta_{m}\left| a_{m} \right|^{2}+\gamma_{m}\left| a_{m} \right|^{2}, (S2a)$$

$$\hat{F}\left( a_{s} \right)=-\frac{1}{2}l_{s}+jD_{s}\frac{\partial^{2}}{\partial t^{2}}-j\delta_{s}\left| a_{s} \right|^{2}+\gamma_{s}\left| a_{s} \right|^{2}, (S2b)$$

where $D_{i}$ is the net dispersion, $\delta_{i}$ is the SPM coefficient and $\gamma_{i}$ is the SAM coefficient. The second and third terms on the right-hand side of Eq. (S1) correspond to the XPM and the stimulated Raman scattering (SRS), respectively, under the assumption that the Raman gain spectrum is broader than the spectra of both the fundamental mode-locked pulse (P_m_) and the Stokes-shifted pulse (P_s_) [S2]. By substituting Eq. (S2) into Eq. (S1), the equation can be rewritten as

$$T_{R}\frac{\partial}{\partial T}a_{m}\left( T, t \right)=\left[ \frac{1}{2}\left( g_{m}-l_{m} \right)+jD_{m}\frac{\partial^{2}}{\partial t^{2}}-j\delta_{m}\left| a_{m}\left( T,t \right) \right|^{2}+\gamma_{m}\left| a_{m}\left( T,t \right) \right|^{2} \right.$$

$$\left. -j\frac{\lambda_{s}}{\lambda_{m}}\delta_{x}\left| a_{s}\left( T,t \right) \right|^{2}-\frac{\lambda_{s}}{\lambda_{m}}\delta_{R}\left| a_{s}\left( T,t \right) \right|^{2} \right]a_{m}\left( T, t \right), (S3a)$$

$$T_{R}\frac{\partial}{\partial T}a_{s}\left( T, t \right)=\left[ -\frac{1}{2}l_{s}+jD_{s}\frac{\partial^{2}}{\partial t^{2}}-j\delta_{x}\left| a_{m}\left( T,t \right) \right|^{2}+\delta_{R}\left| a_{m}\left( T,t \right) \right|^{2} \right]a_{s}\left( T, t \right). (S3b)$$

Here, we have only considered the threshold behaviour of the Stokes-shifted pulse, meaning that we can ignore certain terms, including $\left| a_{s} \right|^{3}$.

The rate equations for the pulse fluence ($W_{i}$) were derived from Eq. (S3) using $\int\left| a_{i}\left( T,t \right) \right|^{2}dt=W_{i}\left( T \right)$. For simplicity, we assume that the pulses around the cavity act as solitons, and the electric field amplitudes are thus written as $a_{i}\left( T, t \right)=A_{i}\mathrm{sech} \left( t/\tau_{i} \right)e^{j\psi T/T_{R}}$, where $\tau_{i}$ is the pulse duration. Therefore, the rate equations for the pulse fluence are

$$T_{R}\frac{d}{dT}W_{m}\left( T \right)=\left[ g_{m}-l_{m}+\frac{2}{3}\frac{\gamma_{m}}{\tau_{m}}W_{m}(T)-\frac{\pi}{4}\frac{\lambda_{s}}{\lambda_{m}}\frac{\delta_{R}}{\tau_{\mathrm{ave}}} W_{s}\left( T \right) \right]W_{m}\left( T \right), (S4a)$$

$$T_{R}\frac{d}{dT}W_{s}\left( T \right)=\left[ -l_{s}+\frac{\pi}{4}\frac{\delta_{R}}{\tau_{\mathrm{ave}}} W_{m}\left( T \right) \right]W_{s}\left( T \right). (S4b)$$

The last terms on the right-hand side were calculated using the approximation $\int\mathrm{sech}^{2} \left( t/\tau_{i} \right)dt\approx\int1/(1+\left( t/\tau_{i} \right)^{2})dt$ to aid in calculation of the time integral. The third term in Eq. (S4a) represents the SAM effect and it does not affect the threshold pulse fluence (Eq. (1)) or the linear increase behaviour of P_s_ near the threshold that is shown in Fig. 1(b), and thus we can ignore this term. As a result, Eq. (2) in the main text was derived.

In the steady state, the emission gain can be given simply as $g_{m}=R/(W_{m}+1/\sigma\tau_{\mathrm{inv}})$ using the rate equation for population inversion, where $R$ is the pump rate, $\sigma$ is the emission cross-section, and $\tau_{\mathrm{inv}}$ is the lifetime of the population inversion [13]. By substituting the gain $g_{m}$ into Eq. (2), a steady-state solution can be derived as follows:

$$W_{m}=\left\{ \begin{aligned} \frac{1}{l_{m}}\left( R-R_{m}^{\left( \mathrm{th} \right)} \right) \left( R_{s}^{\left( \mathrm{th} \right)}>R\geq R_{m}^{\left( \mathrm{th} \right)} \right) \\ \frac{4}{\pi}\frac{\tau_{\mathrm{ave}}}{\delta_{R}}l_{s}\equiv W_{m}^{\left( \mathrm{th} \right)} \left( R\geq R_{s}^{\left( \mathrm{th} \right)} \right), \end{aligned} (S5a) \right.$$

$$W_{s}=\left\{ \begin{aligned} 0 \left( R_{s}^{\left( \mathrm{th} \right)}>R\geq R_{m}^{\left( \mathrm{th} \right)} \right) \\ \frac{1}{l_{s}}\frac{\lambda_{m}}{\lambda_{s}}\frac{\sigma\tau_{\mathrm{inv}}W_{m}^{\left( \mathrm{th} \right)}}{1+\sigma\tau_{\mathrm{inv}}W_{m}^{\left( \mathrm{th} \right)}}\left( R-R_{s}^{\left( \mathrm{th} \right)} \right) \left( R\geq R_{s}^{\left( \mathrm{th} \right)} \right), \end{aligned} (S5a) \right.$$

where $R_{m}^{\left( \mathrm{th} \right)}$ and $R_{s}^{\left( \mathrm{th} \right)}$ are the threshold pump powers; here, $R_{m}^{(\mathrm{th})}=l_{m}/\sigma\tau_{\mathrm{inv}}$ and $R_{s}^{(\mathrm{th})}=R_{m}^{\left( \mathrm{th} \right)}+l_{m}W_{m}^{\left( \mathrm{th} \right)}$. The second solution to Eq. (S4a) is equal to Eq. (1) with the relationship $\delta_{R}\approx g_{R}d/2$, where $g_{R}$ is the Raman gain coefficient and $d$ is the interaction length of the Raman medium. The results from Eq. (S5) are shown in Fig. 1(b). While we did include the SAM effect for the main pulse, the second solution to Eq. (S5a) and the first solution to Eq. (S5b) are exactly the same, and second solution to Eq. (S5b) still shows the linear increase. The threshold peak intensity can be calculated using the relationship $W_{i}=2\tau_{i}{A_{i}}^{2}=2\tau_{i}I_{i}$, where $I_{i}$ is the peak intensity.

**S2. Raman spectra**

Figure S1(a) shows the Raman spectra of Yb:CALGO, Yb:KYW and the Yb:Y_2_O_3_ ceramic when pumped using a 514 nm continuous wave laser. Yb:KYW shows two prominent peaks at 765 cm^−1^ and 905 cm^−1^. The Raman gain coefficients of these peaks were measured to be $g_{R,KYW}\approx3.6 cm/GW$ when pumped at 1064 nm [S3]. Therefore, the Raman gain coefficients of the Yb:Y_2_O_3_ ceramic and the Yb:CALGO are estimated as follows: $g_{R,Y2O3}\approx0.1 cm/GW$ and $g_{R,CALGO}\approx0.01 cm/GW$, respectively. The Raman spectrum of Yb:CALGO shows a broad and flat spectrum. In particular, Yb:CALGO shows unreported peaks in the range from 1000 to 1500 cm^−1^. This led to speculation that we had used a brown-coloured Yb:CALGO crystal that included oxygen defects [S4]. The Raman spectrum of Yb:CALGO has a strong background, as shown in Fig. S1(b). This may represent the emission from a colour centre, and thus we can subtract the background via polynomial curve fitting. After subtraction of the background, the convolution of the Raman spectrum and the main spectrum (P_m_) was calculated.


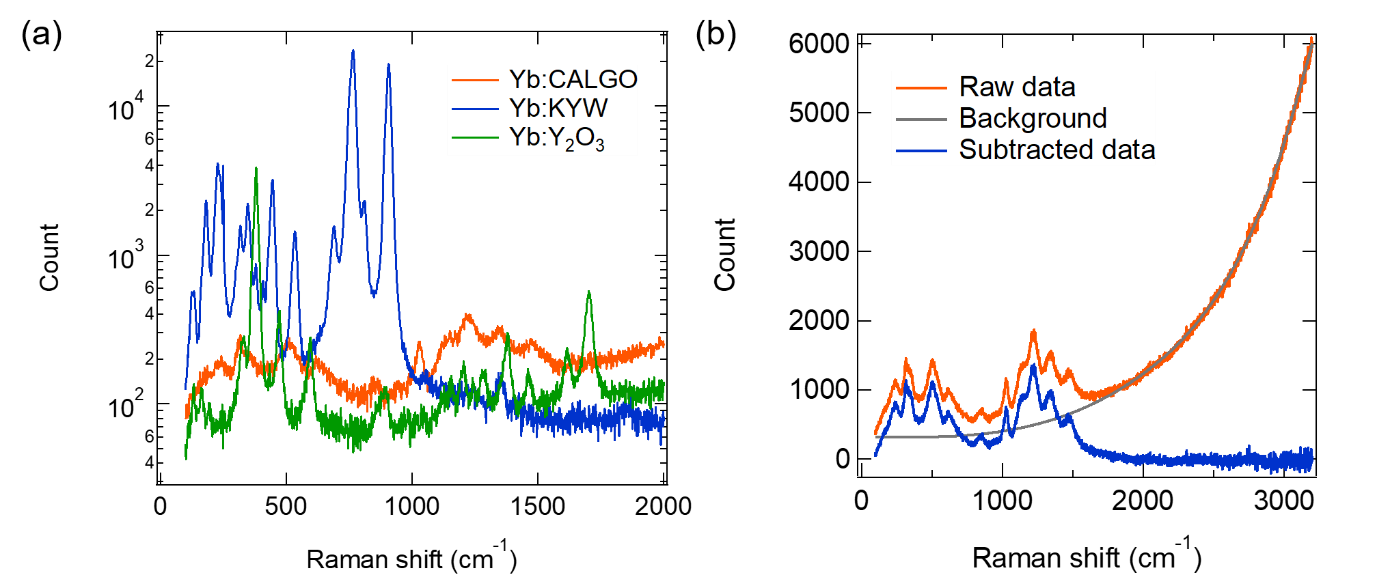


**Figure S1|Raman spectra.** **a.** Raman spectra of Yb:CALGO (orange), Yb:KYW (blue) and Yb:Y_2_O_3_ (green) when pumped using a 514 nm CW laser. **b.** Subtraction of the background from the Raman spectra for Yb:CALGO.

**S3. Kelly sideband**

The centre frequencies of the Kelly sidebands were calculated using the following equation [S5]:

$$\frac{\beta_{2}}{2}\left( \omega-\omega_{0} \right)^{2}+\frac{\beta_{3}}{6}\left( \omega-\omega_{0} \right)^{3}+\frac{\beta_{2}}{2}\frac{1}{\tau_{0}^{2}}=2\pi m. (S6)$$

where $\omega$ indicates the angular frequency, $\omega_{0}$ is the centre frequency of the pump pulse, $\tau_{0}$ is the pulse duration, $m$ is an integer, and $\beta_{2}$ and $\beta_{3}$ are the group delay dispersion (GDD) and the third-order dispersion (TOD), respectively. Table S1 shows the Kelly sideband frequencies for the Yb:CALGO oscillator that were calculated using parameters $\beta_{2}=-450 fs^{2}$, $\beta_{3}=14000 \mathrm{fs}^{3}$ and $\tau_{0}=68 \mathrm{fs}$. The zero-order Kelly sideband ($m=0$) corresponds to the wavelength of P_k_ in Fig. 2(c). In addition, P_k_ showed a red-shift as the bandwidth of the fundamental mode-locked spectrum increased. This behaviour is explained by Eq. (S6). The calculated higher order Kelly sidebands ($m=2, 1, -1, -2$) cannot explain the other peaks shown, such as P_s1_ and P_s2_.

**Table S1| Calculated peak Kelly sideband wavelengths in Yb:CALGO laser**

| m | Wavelength (nm) |
| --- | --- |
| -2 | 1190 |
| -1 | 1165 |
| 0 | 1120 |
| 1 | 986 |
| 2 | 969 |

**S4. Assignment of Raman peak frequencies**

The wavelengths of P_s1_ and P_s2_ for the Yb:CALGO laser are not dependent on the pump power, as shown in Fig. 2(c). It was speculated that the wavelengths of these peaks were determined by the cavity specifications, such as the net reflectivity or dispersion. To verify this hypothesis, we shifted the net reflectivity and net dispersion spectra slightly by varying the bow-tie cavity angle. Figure S2(a) shows the experimental concept. If we vary the cavity angle, the reflection angles at the dielectric mirrors also change, meaning that the reflectivity and the dispersion spectrum can shift slightly. Figure S2(b) shows the output spectra for various angles. The main peak (P_m_) does not change its wavelength, but the Stokes-shifted peaks (P_s1_ and P_s2_) change their wavelengths because the main peak wavelength is largely determined by the emission gain, while the wavelengths of the Stokes-shifted pulses are determined by the GDD or the loss.


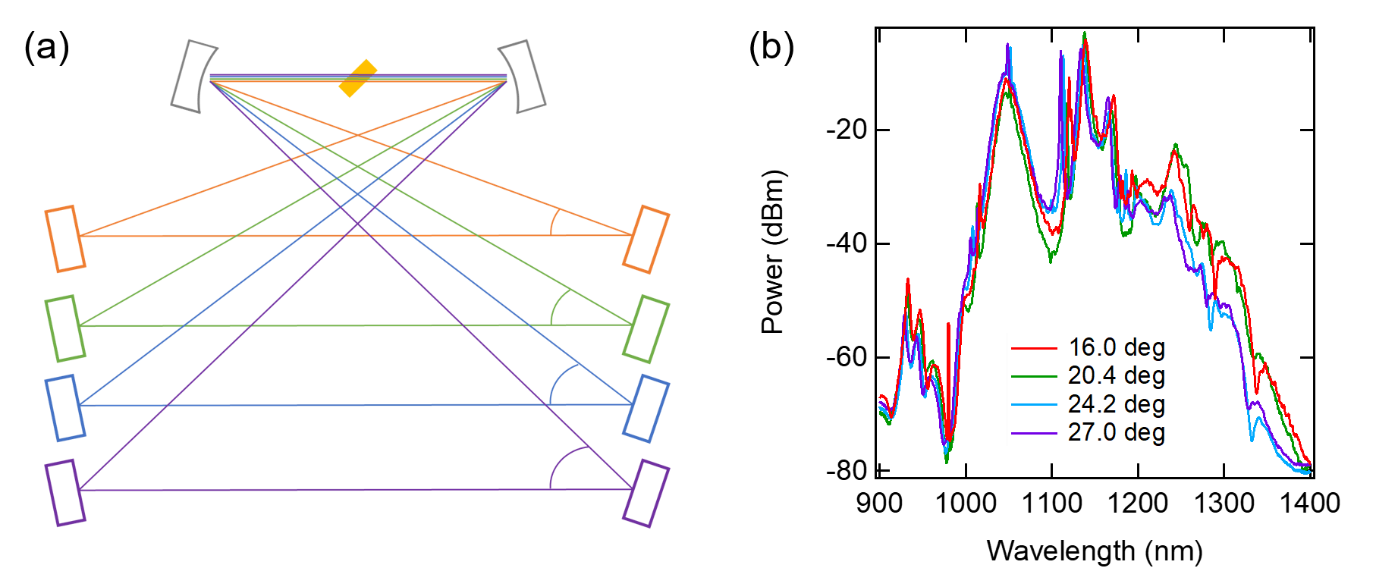


**Figure S2| Cavity angle dependence of output spectra.** **a.** Conceptual figure. **b.** Output spectra of Yb:CALGO laser at various cavity angles.

Figure S3(a) and S3(b) shows the cavity net reflectivity and the GDD, respectively, along with the output spectrum. We see that the three mode-locked peaks (P_m_, P_s1_ and P_s2_) have similar values of the GDD $\beta_{2}\approx-500 fs^{2}$. This may be because of the synchronous pumping nature of the Raman-assisted broadband mode-locked laser.


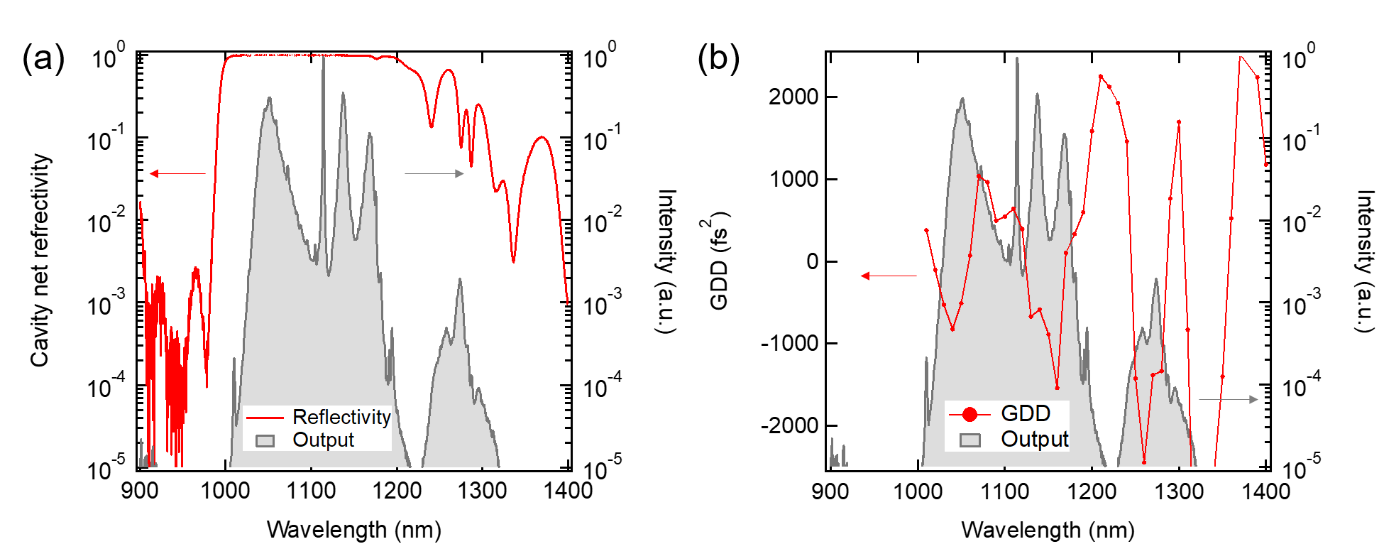


**Figure S3| Cavity net reflectivity and GDD.** **a.** Cavity net reflectance with the Yb:CALGO laser output spectrum. **b.** Cavity net GDD with the Yb:CALGO laser output spectrum.

**References**

S1. Namiki, S., Ippen, E. P., Haus, H. A. & Charles, X. Y. Energy rate equations for mode-locked lasers. *JOSA B* **14**, 2099-2111 (1997).

S2. Headley, C. & Agrawal, G. P. Unified description of ultrafast stimulated Raman scattering in optical fibers. *JOSA B* **13**, 2170-2177 (1996).

S3. Kaminskii, A. A., *et al*. High efficiency nanosecond Raman lasers based on tetragonal PbWO4 crystals. *Opt. Commun.* **183**, 277-287 (2000).

S4. Hu, Q., *et al.* The origin of coloration of CaGdAlO 4 crystals and its effect on their physical properties. *Cryst. Eng. Comm*. **19**, 537-545 (2017).

S5. Curley, P. F., Spielmann, C., Brabec, T., Krausz, F., Wintner, E. & Schmidt, A. J. Operation of a femtosecond Ti: sapphire solitary laser in the vicinity of zero group-delay dispersion. *Opt. Lett.* **18**, 54-56 (1993).
